# Electrophysiological evidence for the enhancement of gesture-speech integration by linguistic predictability during multimodal discourse comprehension

**DOI:** 10.3758/s13415-023-01074-8

**Published:** 2023-02-23

**Authors:** Florian Hintz, Yung Han Khoe, Antje Strauß, Adam Johannes Alfredo Psomakas, Judith Holler

**Affiliations:** 1 Max Planck Institute for Psycholinguistics, Nijmegen, NL The Netherlands; 2 Deutscher Sprachatlas, Philipps University of Marburg, Marburg, Germany; 3 Center for Language Studies, Radboud University, Nijmegen, NL Netherlands; 4 University of Konstanz, Konstanz, DE Germany; 5 Donders Institute for Brain, Cognition & Behaviour, Radboud University, Nijmegen, Netherlands

**Keywords:** Multimodal communication, Language comprehension, Gesture-speech integration, Iconic co-speech gestures

## Abstract

In face-to-face discourse, listeners exploit cues in the input to generate predictions about upcoming words. Moreover, in addition to speech, speakers produce a multitude of visual signals, such as iconic gestures, which listeners readily integrate with incoming words. Previous studies have shown that processing of target words is facilitated when these are embedded in predictable compared to non-predictable discourses and when accompanied by iconic compared to meaningless gestures. In the present study, we investigated the interaction of both factors. We recorded electroencephalogram from 60 Dutch adults while they were watching videos of an actress producing short discourses. The stimuli consisted of an introductory and a target sentence; the latter contained a target noun. Depending on the preceding discourse, the target noun was either predictable or not. Each target noun was paired with an iconic gesture and a gesture that did not convey meaning. In both conditions, gesture presentation in the video was timed such that the gesture stroke slightly preceded the onset of the spoken target by 130 ms. Our ERP analyses revealed independent facilitatory effects for predictable discourses and iconic gestures. However, the interactive effect of both factors demonstrated that target processing (i.e., gesture-speech integration) was facilitated most when targets were part of predictable discourses and accompanied by an iconic gesture. Our results thus suggest a strong intertwinement of linguistic predictability and non-verbal gesture processing where listeners exploit predictive discourse cues to pre-activate verbal and non-verbal representations of upcoming target words.

## Introduction

Human language is inherently multimodal, consisting of words, sentences as well as the plethora of visual bodily signals that accompany linguistic elements (e.g., Bavelas, 2022; Enfield, 2009; Holler & Levinson, 2019; Kendon, 2004; McNeill, 1992; Vigliocco et al., 2014). The hands are one of the main articulators contributing to co-speech visual communication. Manual gestures are frequent during speaking and carry a substantial amount of semantic (McNeill, 1992; Holler & Beattie, 2003; 2002; Holler et al., 2009; Rowbotham et al., 2014; Hostetter, 2011; Kendon, 2000; Kita & Özyürek, 2003) and pragmatic information (Bavelas et al., 1992, 1995; Kendon, 2004). Moreover, they play a significant role during language comprehension. Especially iconic gestures—those movements of the hands and arms that depict actions, objects and their attributes (McNeill, 1992)—are processed in brain regions dedicated to linguistic and semantic processing (left IFG, pSTS, MTG), and are integrated with speech during comprehension (Kelly et al., 2004; Kelly et al., 2010a; Willems et al., 2007, 2009; Wu and Coulson, 2005; Wu & Coulson, 2010; Holle & Gunter, 2007; Holle et al., 2008; Green et al., 2009; Dick et al., 2009, 2014, see Kandana Arachchige et al., 2021 for review). These findings have substantially corroborated the notion that manual co-speech gestures form an integral part of human language.

One aspect that is considerably less well researched is the precise interface between speech and gesture during comprehension. Kelly and colleagues (2010b) proposed that the integration of speech and iconic gestures is obligatory. That is, iconic gestures are assumed to be readily integrated with speech, even when we try to disregard them. However, the integration of gestures with speech is influenced by verbal and non-verbal factors. These factors include the semantic congruency between the two modalities (Kelly et al., 2010b), the speaker’s intentional stance (Kelly et al., 2007) and co-occurring visual signals, such as speaker gaze direction (Holler et al., 2014, 2015) and body orientation (Nagels et al., 2015; He et al., 2020, i.e., indicating that a speech-gesture utterance is intended for another recipient).

Similarly, the integration of gestures is influenced by properties of the speech they accompany. A particularly critical aspect in this respect is the extent to which speech may or may not be predictive of upcoming information. Predictive processing has become a major focus in (neuro)cognitive investigations of language and turned into a core feature of theoretical frameworks and processing models (Altmann & Mirkovic, 2009; Brouwer et al., 2017; Huettig, 2015; Huettig et al., 2022; Kuperberg & Jaeger, 2016; Pickering & Gambi, 2018). Despite some debate about the representational levels at which predictive language processing happens (Nieuwland et al., 2020, 2018), an impressive body of experimental work has accumulated suggesting that comprehenders exploit predictable information in the speech signal in the service of facilitating comprehension. Importantly, the vast majority of studies that motivated such theories were done in uni-modal contexts (e.g., spoken or written language). However, since face-to-face communication is the most frequent form of language use (Levinson & Holler, 2014), they fall short of describing the whole picture.

There are a few exceptions. For example, Fritz et al. (2021) investigated whether gestures that temporally precede target words with which they are semantically affiliated are integrated into discourse models. The discourse contexts preceding the target words were manipulated to be constraining or non-constraining. Their results suggested that even gestures that precede their semantic affiliates are integrated into predictable discourse models, as evidenced by a P600 as measured from target word onset. Specifically, the authors interpreted this ERP component as indexing that gesture meaning may have initially remained ambiguous (i.e., after early gesture presentation) and that listeners re-interpreted the discourse meaning “as more relevant information enter[ed] the discourse” (Fritz et al., p. 15)—after target word onset. They did not, however, observe an N400 effect—neither at the point of gesture presentation, nor at the point of target word presentation—suggesting that gesture meanings could not be readily integrated with the discourse models, nor could they be mapped onto the concepts of the semantic affiliates (i.e., spoken target words). The authors conjectured that this was probably due to the meaning of the gestures being too ambiguous and the predictable discourses being only moderately constraining.

In a related study, Zhang et al. (2021) used seminaturalistic stimuli of an actress producing two-sentence passages extracted from the British National Corpus (University of Oxford, 2007) and the BBC script library. While producing the passages, the actress was allowed to gesture freely. Zhang et al. quantified the predictability of individual words in the passages using ‘surprisal’ (Shannon, 1949)—an information-theoretic measure based on co-occurrence frequency that has been shown to modulate word reading times (Smith & Levy, 2013) and the words’ N400 amplitudes (Frank et al., 2015; Michaelov et al., 2022). As to be expected, their analyses showed that words with higher surprisal values (i.e., less expected words) elicited larger N400 amplitudes than words with lower surprisal values. Moreover, words that were accompanied by meaningful (i.e., iconic) gestures also elicited reduced N400 amplitudes compared with words in the absence of an iconic gesture. Crucially, the authors also observed an interaction between predictability and gesture presence such that high surprisal words elicited larger reductions in N400 amplitude when meaningful gestures were present compared with low surprisal words. That is, iconic gestures made the words they accompany “less surprising,” which may relate to the general tendency that iconic gestures start slightly earlier than their semantic affiliates (ter Bekke et al., 2020). Co-occurring gestures thus modulated the predictability of words as indicated by the N400 amplitude reduction in Zhang et al.’s (2021) study. This is an interesting finding, which underlines the deeply multimodal nature of human language processing in face-to-face contexts.

The question that we addressed in the present study is the flipside of the above, namely whether the predictability of the preceding discourse context leading up to the occurrence of a gesture influences the integration of that gesture with the word it accompanies. While Fritz et al. (2021) tested for the effect of constraining preceding discourse on gestures, they did so for nonsynchronous gestures only. Their study was not intended to measure the effect of the predictability of preceding discourse on the semantic integration of speech and gestures when they co-occur. While Zhang et al. (2021) did focus on linguistic predictability and the semantic integration of co-occurring speech and gestures, they did not systematically manipulate the predictability of the preceding discourse. Rather, they measured the simple and interactive effects of gesture presence (present vs. absent) on individual words’ N400 amplitude in the unfolding sentences. The question of how a core feature of human language processing—i.e., the extent to which an unfolding discourse that does or does not constrain the prediction of upcoming words influences the integration of gestures accompanying those words—therefore necessitates further enquiry.

### Present study

We investigated this question by asking participants to observe and listen to an actress producing target words (denoting objects, e.g., kangaroo), which were either preceded by a highly constraining or nonconstraining discourse context (assessed in a cloze probability rating task, Taylor, 1953), rendering the target word predictable or not. Thus, we compared how the same target word is processed when embedded in a predictable and in a nonpredictable linguistic context. Moreover, the target words were either accompanied by an iconic gesture depicting the object denoted by the target word or by a noncommunicative biological movement (e.g., a scratching movement). We opted for such a contrast (rather than a plain comparison of presence/absence, i.e., featuring no movement in the absence condition) to accommodate effects of motion processing on ERPs elicited by the target words.

As has been demonstrated numerous times (Nieuwland et al., 2018, 2020; Van Berkum et al., 2005), we expected participants to exploit discourse information in the constraining discourse condition to generate predictions about the upcoming target words. Therefore, compared with the nonpredictable condition, target word processing should be facilitated in the predictable discourse condition, as reflected in reduced N400 amplitudes.

Based on the study by Zhang et al. (Zhang et al., 2021; see also Willems et al., 2007, 2009), we also hypothesized that the presence of meaningful gestures presented in close proximity to the target words would facilitate target word processing, irrespective of the preceding discourse being constraining or nonconstraining. The reason is that iconic gestures provide an additional modality through which listeners can access the target concept. Thus, we expected facilitated processing for target words accompanied by iconic gestures compared with target words accompanied by control movements to be reflected in reduced N400 amplitudes.

The main focus of our analyses was, however, on the interaction between discourse predictability and the presence of meaningful gestures. We expected that discourse contexts that are highly predictive of a target word enhance the interpretability of iconic gestures and thus facilitate their integration. For example, if you listen to someone speak about local animals one typically encounters in Australia, you will be generating predictions about them mentioning a “kangaroo” as kangaroos are part of most people’s generalized knowledge about Australia (Hintz et al., 2020; Metusalem et al., 2012). Activating knowledge about kangaroos through spoken discourses may include visual information (Huettig et al., 2022), which should facilitate the integration of the iconic gesture with the target word. According to this account, we expected to observe processing differences (reflected in differences in N400 amplitudes) between target words embedded in predictable and nonpredictable discourses, accompanied by iconic gestures. That is, since in the nonpredictable condition listeners could not generate predictions about the upcoming target word, they also could not preactivate visual information that would facilitate iconic gesture-target word integration.

Importantly, based on the results by Zhang et al. (2021), an alternative prediction is possible. Recall that Zhang and colleagues observed that the presence of meaningful gestures in their paradigm elicited larger reductions of N400 amplitude in high-surprisal words (i.e., lower predictability) than in low-surprisal words (i.e., higher predictability). That is, the presence of meaningful gestures mitigated the efforts associated with processing words of lower compared with higher predictability. Against this background, one could hypothesize the opposite pattern concerning the interaction between discourse predictability and the presence of meaningful gestures: Processing of target words embedded in nonpredictable discourses may benefit more from the presence of meaningful gestures than target words embedded in predictable discourses—possibly because the gain in activating the target concept is larger in the nonpredictable condition.

Finally, we did not expect these patterns to occur for the nongestural movements, because they should be segregated and disregarded during the integration process due to their noncommunicative nature (with the exception of some very early integration attempts perhaps when the movement has just begun and could still evolve to be either gestural or nongestural in nature).

## Method

### Participants

The sample size was determined a priori. Sixty-three, healthy, right-handed, native speakers of Dutch (46 females) were recruited from the subject pool of the Max Planck Institute for Psycholinguistics. None of them had hearing problems or neurological or developmental impairments. Participants’ vision was normal or corrected to normal. None of the participants had participated in any of the pretests. All participants provided written consent before taking part in the experiment and were paid 18€ as compensation. The study was approved by the Ethics Board of the faculty of Social Sciences at Radboud University and complied with the Declaration of Helsinki. Three participants were excluded from all statistical analyses (see below for details), due to excessive data loss after pre-processing (N = 2) and due to poor performance on the comprehension questions (N = 1). The final dataset consisted of 60 participants (mean age = 24.3, range 18–34 years, standard deviation [SD] = 3.47; 44 females). Testing for the EEG experiment started at the beginning of 2020 and was interrupted by the first Covid-19-related lockdown in the Netherlands after participant #35. Testing resumed in April 2021 and was completed in July of the same year.

### Materials

The stimulus set consisted of 80 concrete target nouns (mean Zipfian frequency = 3.92, SD = 0.90, range = 2.06–6.47, Keuleers et al., 2010; mean prevalence = 0.99, SD = 0.02, range = 0.91-1, Keuleers et al., 2015), which were embedded in 160 contexts. The contexts comprised short Dutch discourses consisting of two sentences, ending in the target nouns. In 80 discourses, the target word could be predicted from the preceding context; in the remaining 80, the target word could not be predicted. Each target word was paired with an iconic gesture that depicted the target noun and with a noniconic control movement that was unrelated to the target word, yielding a total of 320 unique stimuli. Stroke onset in both types of gestures was timed to start 130 ms before target word onset (Drijvers & Özyürek, 2018, Fig. 1, for an example), meaning that gesture and target word were to a large extent processed simultaneously.

**Fig. 1:**
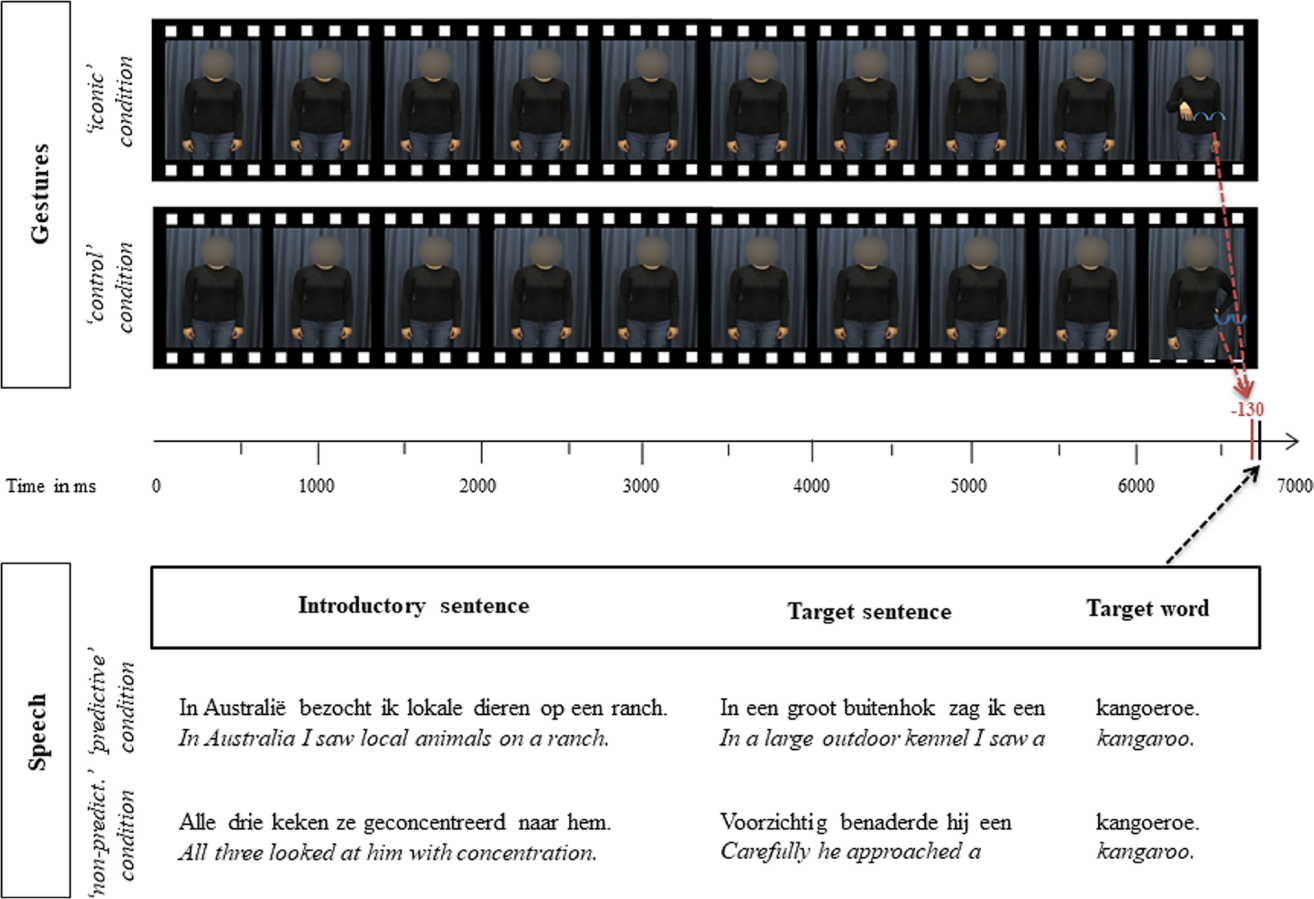
Overview of the trial structure in the different conditions

Video recordings of the stimuli were made in the video recording laboratory of the Max Planck Institute for Psycholinguistics. A female, native speaker of Dutch was videotaped while producing the spoken discourses using normal intonation and a regular speaking rate. Next to producing a discourse, she executed iconic and control movements. The speaker wore clothes in a neutral dark color and stood in front of a unicolor curtain. She was positioned to be in the center of the screen. At sentence onset, her arms were hanging casually by her sides. She produced the gesture at a point in time that felt natural to her, always close to the target word, but no specific instructions on the timing were given (i.e., the actress was blind to the goal of the present study). At least three versions of each stimulus were recorded. From these three versions, the best recording was selected based on the naturalness of speech and gesture, consistency of speech and gesture across different conditions, and quality of the recording (e.g., absence of background noise, video recording artefacts, etc.). We used ELAN (Version 4.1.2, Wittenberg et al., 2006) to annotate the onset and offset of several events in the video: the target word, the gesture phrase (i.e., from the first to the last frame in which manual movement could be observed that belonged to a gesture), and the gesture stroke phase (the most meaning-bearing part of the gesture; Kita et al., 1998). The video recordings were further edited using Adobe After Effects© to add a mask blurring the speaker's face, such that facial movements and expressions were not visible. Finally, we used ffmpeg© to shift the video track of the stimuli recordings relative to the audio track such that the onset of the gesture stroke preceded the onset of the spoken target by 130 ms in every stimulus video. The spoken target words were on average 646-ms long (SD = 173, range = 289-1,230); gesture strokes were on average 653-ms long (SD = 215, range = 240–1,320). Gesture strokes and target words overlapped by on average 367 ms, ranging from −115 ms (no overlap) to 1,042 ms. Target word onset occurred on average after 6,704 ms (SD = 1,155 ms, range = 4,374-10,336 ms).

### Rating studies

We conducted two web-based sentence completion studies to assess the cloze probability of the target words in the predictable and nonpredictable discourses (Taylor, 1953). Moreover, a lab-based rating study was run to assess how unambiguously the iconic gestures represented the target nouns in the absence of speech (i.e., when presented outside of the spoken discourse context).

#### Sentence completion

Both sentence completion studies were implemented in LimeSurvey (LimeSurvey GmbH). The participants read the discourses up until and including the determiner preceding the target word and were instructed to fill in the word they thought would be the most likely continuation of the running sentence. Thirty participants took part in the first rating study involving 80 predictive contexts (22 females, M = 26.2 years, SD = 3.5 years, range = 21–33 years); another 31 participants took part in the second rating study involving 80 nonpredictive contexts (18 females, M = 24.0 years, SD = 4.0 years, range = 18–33 years). Participants’ responses were coded as “match” in case the word in question was provided. In the case of a nontarget response, the pairwise semantic distance to the target word was calculated by using the Dutch version of Snaut (Mandera et al., 2017). The semantic distance values were then converted to similarity values by subtracting them from 1. Finally, the cloze probability for each target word was calculated by summing up “matches” (i.e., value of 1) and similarity values for nontarget responses (value between 0 and 1) and by dividing this sum by the number of participants who responded. For the predictable contexts, the average cloze probability was 0.85 (SD = 0.13, range = 0.51–1). For the nonpredictable contexts, the average cloze probability was 0.

#### Gesture iconicity

Thirty-two participants (23 females, M = 23 years, SD = 2.9 years, range = 19–31 years) took part in the laboratory-based iconic gesture interpretability rating study, which was implemented in Presentation (version 20.0; Neurobehavioral Systems Inc.). On each trial, the participants first saw a video recording of one of the 80 iconic gestures without audio. They were then asked to provide a maximum of three guesses (nouns) of what the gesture might denote (free entry format, interpretability measure). Finally, they were shown the target word and asked to rate the compatibility of the just-seen gesture and the target word it depicted, using a scale ranging from 1 (incompatible) to 7 (fully compatible, compatibility measure). On average, the probability of the target word being among the three words provided by participants was 0.38 (SD = 0.32, range = 0–1); the mean probability of the target word being the first guess was 0.30 (SD = 0.30, range = 0–1). The average compatibility rating was 5.16 (SD = 1.24, range = 1.75–7), indicating good compatibility.

Taken together, the three rating studies confirmed the suitability of the stimuli for the purposes of the present study. The cloze probability studies demonstrated that predictable and nonpredictable items were classified appropriately. The gesture rating study demonstrated that the iconic gestures we selected to embody the target words were well interpretable when presented on their own.

### Experimental design and lists

We used a 2 (gesture type: iconic vs. control) x 2 (discourse predictability: predictable vs. nonpredictable) mixed design, with repeated measures on the first factor. Thus, each participant either heard predictable or nonpredictable discourses while being presented with both iconic and control movements. Experimental lists were constructed such that the same target word did not appear twice on one list. Half of the trials on each list belonged to the iconic-gesture condition; the other half were control-gesture trials. On the basis of the four resulting experimental lists, pseudo-randomized versions were created before testing using the program “Mix” (van Casteren & Davis, 2006). The pseudo-randomization allowed a maximum of three repetitions of the same gesture type (i.e., iconic or control). The experimental trials on each list were preceded by the same two practice trials. In an alternating fashion, participants were assigned to predictable and nonpredictable conditions, such that the total number of participants on each list was balanced.

### Procedure

Following the general informed-consent procedure, participants were fitted with an EEG cap. During EEG recording, participants were seated in front of a computer monitor, with speakers placed on either side. Participants were seated in a sound-attenuating and electrically shielded booth. The stimuli were presented full screen on a 23-inch monitor operating at a 1,920 x 1,080 pixels native resolution, using the Presentation software (version 20.0; Neurobehavioral Systems, Inc.). Participants were assigned to either the predictable or the non-predictable discourse condition. Twenty of the 80 experimental trials (appearing in a pseudo-random order with variable intervals in between) were followed by a yes/no question to ensure that participants were looking at the computer screen during the experiment. On such a catch trial, a red asterisk was presented in the center of the screen, on top of the video, 200 ms after the offset of the spoken target. The asterisk was presented for 500 ms. Participants were instructed to indicate whether they saw the red asterisk or not by pressing the “Z” key on the keyboard to provide a no-response or by pressing the “M” key to provide a yes-response. The asterisk was presented on half of the catch trials. After every 20 trials, participants were able to take a short, self-timed break before continuing the experiment.

### EEG data recording

Participants’ EEG was recorded throughout the whole test session using BrainVision Recorder software (version 1.20.0401; Brain Products GmbH), at a sampling rate of 1,000 Hz, using a time constant of 8 s (0.02 Hz) and high cutoff of 100 Hz in the hardware filter. The EEG signal was recorded from 27 active scalp electrodes (Fz, FCz, Cz, Pz, Oz, F3/4, F7/8, FC1/2, FC5/6, C3/4, CP1/2, CP5/6, T7/8, P3/4, P7/8, O1/2), mounted in an elastic cap (ActiCAP) according to the 10-20 convention. The EEG signal was recorded with an online reference to the left mastoid. Additionally, activity was recorded at the right mastoid and at four bipolar electrooculogram (EOG) channels (two horizontal and two vertical). The ground electrode was located on the forehead. Triggers were time-locked to both gesture stroke onset and target word onset.

### Data pre-processing

We included participants in the data pre-processing whose accuracy on the yes/no comprehension questions was 80% or higher. This criterion led to the exclusion of one participant, who had scored 75%.

For the pre-processing of the EEG data, we used BrainVision Analyzer (version 2.2.0.7383, Brain Products GmbH). First, the data were re-referenced to the average of left and right mastoid channels. Then, they were filtered using a Butterworth IIR filter, with 0.01 Hz as a low cutoff and 30 Hz as a high cutoff. Next, the continuous data were segmented into epochs, ranging from −500 ms to 1,000 ms, relative to the onset of the target word. This step was followed by ocular artifacts correction (Gratton et al., 1983). As the fifth step, semiautomatic artifact rejection was applied. More specifically, BrainVision Analyzer highlighted trials where channel values exceeded ±50 μV, which were then examined and then kept or rejected on an individual basis. Importantly, only participants were included in the final analysis, who retained at least 60 of 80 trials (75%). Applying this criterion led to the exclusion of two participants—both retained only 73% of trials. For the remaining 60 participants, a total of 261 trials was excluded (5.44%). Trial exclusions were similar in iconic-gesture (2.15%) and control-gesture (2.20%) conditions.

As the final EEG data pre-processing step, baseline correction was applied using a 200-ms window (−500 ms to −300 ms, relative to the onset of the target word, before gesture stroke onset). The average accuracy on the comprehension questions across all 60 included participants was 0.99 (SD = 0.02, range = 0.9-1).

### Data analysis

We created grand-average ERP plots based on single-subject averages for each of the four conditions (predictable-iconic, predictable-control, nonpredictable-iconic, nonpredictable-control). To statistically examine how linguistic predictability modulated the integration of speech and iconic gestures, we used a twofold approach. We first analyzed the data in a similar way as Zhang et al. (2021) did. That is, at the trial-level, we averaged activities of Cz and Pz electrodes (electrodes commonly associated with the N400, Kutas & Federmeier, 2011) during the N400 period (300-600 ms after target word onset)—the same time window as used by Zhang et al. (2021)—and submitted these to a linear mixed-effects model analysis in R (version 4.1.2; R Core Team), using the lme4 package (version 1.1-30; Bates et al., 2014). In total, 60 participants contributed 4,539 data points. The model contained Discourse (predictable = 0.5 vs. nonpredictable = −0.5) and Gesture type (iconic = 0.5 vs. control = −0.5) as (contrast-coded) fixed factors, as well as their interaction. Participant and Item were included as random effects. We added random intercepts to both random effects as well as random slopes for Gesture type (the within-participants condition) by Participant. The formula to call up the model was the following:

$$(1)\ \textrm{lmer}\ \left(\textrm{N}400\ \textrm{amplitude}\sim {\textrm{Gesture}}^{\ast }\ \textrm{Discourse}+\left(1+\textrm{Gesture}\ |\ \textrm{Participant}\right)+\left(1+\textrm{Discourse}\ \textrm{predictability}\ |\ \textrm{Item}\right),\textrm{data}=\textrm{data},\textrm{control}=\textrm{bobyqa}\right)$$

Complementing this literature-based analysis, we used cluster-based permutation (CBP) testing, as implemented in Fieldtrip (Oostenveld et al., 2011), for further exploration of the data. CBP is a nonparametric randomization technique that identifies clusters of significant differences between conditions in time and space while minimizing the multiple-comparisons problem (Maris & Oostenveld, 2007). This approach allowed for analyzing the data without selecting a priori time windows and/or sets of electrodes. Specifically, CBP enabled us to find main and interaction effects beyond Cz and Pz electrodes and outside the predefined time region(s).

To that end, we conducted three cluster searches: one for the main effect of Discourse; one for the main effect of Gesture; and one for the interaction between both factors. For the main effect of Discourse, the between-participants manipulation, we calculated event-related potentials for each participant by averaging over iconic and control movements. We then compared the group that was presented with predictable contexts (N = 30) with the group that was presented with nonpredictable contexts (N = 30) with an independent samples t-test searching for clusters between 200 and 1,000 ms after target word onset. A Monte-Carlo permutation (1,000 random assignments of participants to one group or the other, recalculating the independent *t*-tests) estimated type I-error controlled cluster significance probabilities (α = 0.025).

For the main effect of Gesture type, manipulated within participants, single-trial time-domain EEG data were submitted to a multi-level or “random effects” statistics approach (Strauß et al., 2022). On the first level (i.e., for each individual separately), massed independent samples *t*-tests were calculated to compare iconic versus control movements. Uncorrected *t*-values were obtained for all time-channel bins. On the second (i.e., group) level (N = 60), *t*-values were z-scored for better comparability between participants and were tested against zero in a two-tailed dependent samples *t*-test searching for clusters between 200 and 1,000 ms. Using Monte-Carlo nonparametrical permutation (1,000 randomizations), type I-error controlled cluster significance probabilities (α = 0.025) were estimated.

For interaction effects involving Discourse and Gesture, we took the individual t-contrast maps calculated in the previous analysis, where single-trial time series of iconic versus control movements were compared in each participant. We then compared z-scored t-maps of the two discourse groups with each other (N = 30 each) using independent samples *t*-tests searching for clusters between 200 and 1,000 ms. A Monte-Carlo permutation (1,000 random assignments of participants to one group or the other) estimated type I-error controlled cluster significance probabilities (α = 0.025).

## Results

**Fig. 2:**
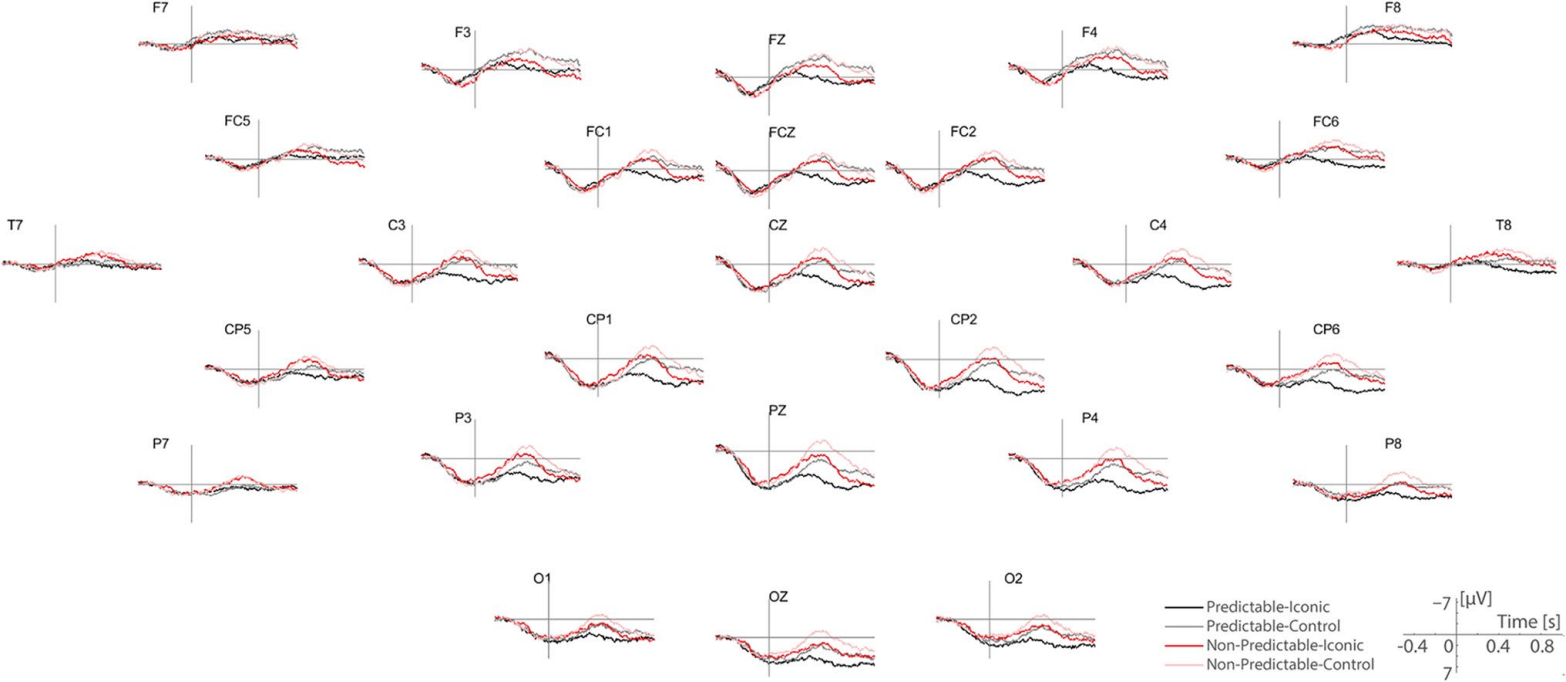
Grand average ERPs elicited by the target words in the four conditions. Time zero refers to the spoken onset of the target word. Negative voltage is plotted up

In Fig. 2, we present the grand-average ERPs for the four experimental conditions and for all recorded electrodes. As shown, the activity was highly comparable during the baseline period (i.e., before gesture stroke onset at 130 ms before target word onset) across all electrodes. Approximately 200 ms after target word onset, frontocentral and centroparietal electrodes showed a difference between predictable and nonpredictable discourse conditions, with nonpredictable discourse conditions eliciting more negative activity. This difference was sustained until approximately 600 ms after target word onset where visual inspection suggests a cross-over of the nonpredictable discourse–iconic gesture condition and the predictable discourse–control movement condition.

**Fig. 3:**
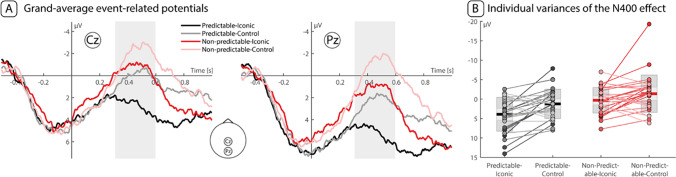
**A**. Grand-average ERP plots for Cz and Pz electrodes. Area shaded in gray represents the N400 window (300-600 ms after target word onset). **B**. Extracted N400 data averaged over Cz and Pz. Boxplots represent the mean and standard error of the mean within each condition. Dots represent individuals and the lines illustrate the condition difference for this participant

Figure 3A zooms in on the activity elicited by Cz and Pz electrodes, on which we conducted our planned analysis. During the N400 window (300-600 ms after target word onset), we observed the following pattern: Predictable target words paired with iconic gestures elicited the lowest N400 amplitude. The predictable discourse–control movement and the nonpredictable discourse–iconic gesture conditions elicited very similar activities to one another. The predictable discourse–control movement and the nonpredictable discourse–iconic gesture conditions elicited very similar activities to one another. The nonpredictable discourse–control movement condition elicited the most negative amplitude. Figure 3B additionally presents mean and individual participant voltages in the four conditions for the Pz-Cz electrode complex during the N400 time window. This pattern corroborates the intuition that predictable discourses elicited more positive activity than nonpredictable discourses and that iconic gestures elicited more positive activity than control movements across both discourse types.

### N400 analysis (300–600 ms after target word onset)

Our analysis of the N400 time window over Cz and Pz electrodes revealed significant main effects of both Discourse (β = 3.08, 95% confidence interval [CI] = [1.29, 4.87], SE = 0.90, t = 3.42, *p* = 0.001) and Gesture type (β = 2.13, 95% CI = [1.10, 3.16], SE = 0.52, t = 4.10, *p* < 0.001), with predictable target words and iconic gestures eliciting more positive ERPs, compared with nonpredictable target words and control movements, respectively. This model showed no evidence for an interaction between Discourse and Gesture (see Table 1, for an overview of the results). However, given our theoretically motivated predictions, we conducted planned comparisons between (1) the predictable discourse-iconic gesture and the predictable discourse-control movement conditions and between (2) the nonpredictable discourse-iconic gesture and the nonpredictable discourse-control movement conditions, using the “emmeans” package in R. These analyses revealed that iconic gestures elicited more positive ERPs than control movements in both the predictable (emmean = 2.66 (95% CI = [1.22, 4.09]), SE = 0.73, z.ratio = 3.63, *p* < 0.001, Cohen’s d = 0.21) and nonpredictable (emmean = 1.60 (95% CI = [0.16, 3.04]), SE = 0.74, z.ratio = 2.17, *p* = 0.03, Cohen’s d = 0.13) discourses. As the estimated marginal means and effect sizes suggest, the difference was larger in the predictable relative to the nonpredictable discourses.

**Table 1:** Linear mixed-effects model output for the N400 window (300–600 ms after target word onset) over Cz and Pz electrodes

Predictor	β	95% CI	SE	t	*p*
Intercept	1.05	[0.07, 2.02]	0.49	2.14	0.036
Discourse predictability	3.08	[1.29, 4.87]	0.90	3.42	0.001
Gesture type	2.13	[1.10, 3.16]	0.52	4.10	<0.001
Discourse x Gesture	1.06	[−1.01, 3.12]	1.04	1.02	0.311

### Cluster-based permutation analysis

**Fig. 4:**
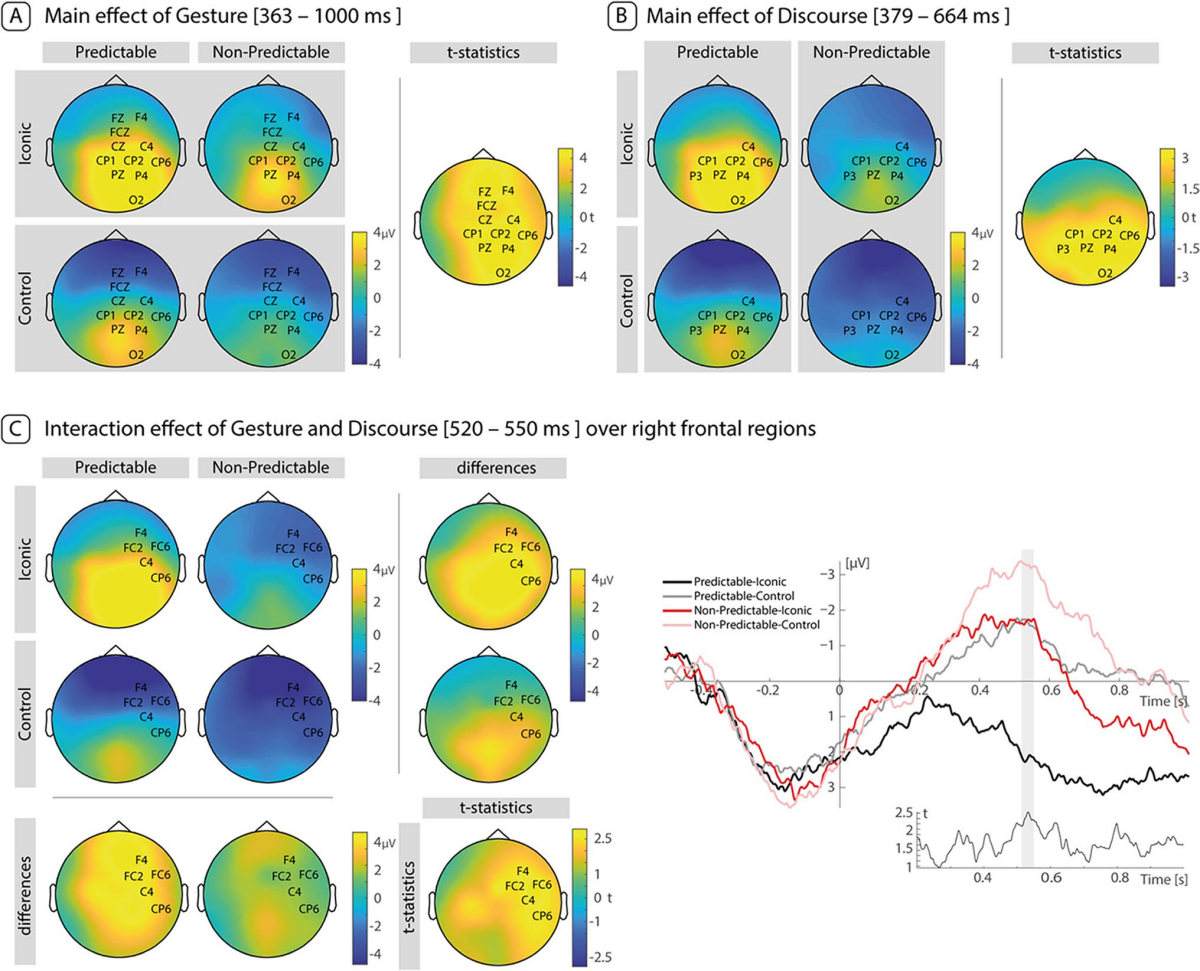
Results of CBP analyses. **A**. Main effect of Gesture. **B**. Main effect of Discourse. Topographies in A and B complement the timelines plotted in Fig. 2 and represent the average over the whole time window, as detected in the cluster-based permutation analysis. **C**. Interaction effect of Gesture and Discourse. Grand-average ERP is plotted for activity elicited on the highlighted electrodes, complemented with the t-values over time. The area shaded in blue highlights the significant time window

The cluster search for main effects of Discourse revealed one positive cluster (*p* = 0.007, T_sum_ = 12,149; Fig. 4B), during the window from 379 ms to 664 ms after target onset. This cluster was distributed over parietal electrodes (strongest effects over C4, CP1, CP2, CP6, P3, Pz, P4, O2) but also included fronto-temporal electrodes: FC1, FC2, FC6, T7, C3, Cz, T8, CP5, P7, P8, O1, Oz). Thus, while the CBP analysis confirmed the results of the planned N400 analysis (involving Cz and Pz electrodes), it additionally showed that the Discourse effect was wide-spread over frontoparietal electrodes, extending well beyond Cz and Pz electrodes.

The cluster search for main effects of Gesture revealed one positive cluster (*p* < 0.0001, T_sum_ = 51,973; Fig. 4A), during the window from 363 ms to 1,000 ms (i.e., the end of the analyzed time period). This cluster, too, was broadly spread over the entire midline (strongest effects over Fz, F4, Cz, C4, FCz, CP1, CP2, CP6, Pz, P4, P8, O2) but also included F7, F3, F8, FC5, FC1, FC2, FC6, T7, C3, T8, CP5, P3, O1, Oz). As for the effects of Discourse, these results are in line with the planned N400 analysis on the Cz-Pz electrode complex but highlight the broadness of the effects in space and time.

The cluster search for interaction effects between Discourse and Gesture revealed one positive cluster (*p* = 0.046, T_sum_ = 646.34; Fig. 4C), during the period from 519 ms to 552 ms over right frontal electrodes (strongest effects over F4, FC2, FC6, C4, CP6) but also included Fz, F8, Cz, T8, FCz, CP2, P4). Because this cluster contained primarily right-frontal electrodes, we did not detect this effect in our N400 analysis, although its temporal locus fell into the N400 time window. We followed up on this finding by extracting the EEG data, averaged over the determined time and regions of interest and submitted them to post-hoc paired *t*-tests that compared between (1) the predictable discourse-iconic gesture and the predictable discourse-control movement conditions and between (2) the nonpredictable discourse-iconic gesture and the non-predictable discourse-control movement conditions. The results show that iconic gestures elicited more positive ERP amplitudes than control movements when presented in predictable discourses (emmean = 3.84 (95% CI [2.38, 5.31]), t(29) = 5.37, *p* < 0.001, Cohen’s d = 0.98), as well as when presented in nonpredictable discourses (emmean = 1.60 (95% CI [0.05, 3.14]), t(29) = 2.12, *p* = 0.043, Cohen’s d = 0.39). However, the differences in predictable discourses were bigger than in nonpredictable discourses (95% CI [0.16, 4.33]), t(58) = 2.16, p < 0.035, Cohen’s d = 0.36).

In summary, our exploratory CBP analyses revealed main effects of discourse predictability and gesture type, with predictable target words eliciting more positive ERPs than nonpredictable target words and iconic gestures eliciting more positive ERPs than control movements. Both main effects had their onset around 350 ms after target word onset. Importantly, we also observed evidence for an interaction between discourse predictability and gesture type during a time window starting 520 ms after target onset. The post-hoc comparisons revealed that iconic gestures paired with predictable and nonpredictable target words elicited more positive effects than the same words paired with control movements.

## Discussion

The present study was designed to test whether linguistic predictability modulates the integration of speech and iconic gestures.^1^ In our analysis that focused on the N400 amplitude we observed a main effect of discourse predictability with facilitated processing when target words were embedded in predictable compared to nonpredictable discourses. Although not our main focus, this finding adds to the growing body of research demonstrating that participants exploit predictive linguistic cues when listening to discourse in the service of facilitating comprehension (Huettig, 2015; Pickering & Gambi, 2018).

Our N400 analysis further revealed a main effect of gesture type, with facilitated processing of target words accompanied by iconic gestures as compared to when they were accompanied by meaningless control movements (e.g., scratching). This is in line with the well-established finding that speech and co-speech iconic gestures tend to be readily integrated during semantic processing (Kelly et al., 2004; Kelly et al., 2010a; Willems et al., 2007, 2009; Wu and Coulson, 2005; 2010; Holle & Gunter, 2007; Holle et al., 2008; Green et al., 2009; Dick et al., 2009, 2014, see Kandana Arachchige et al., 2021 for a review), because iconic gestures form an inherent part of human language (Bavelas, 2022; Enfield, 2009; Holler & Levinson, 2019; Kendon, 2004; McNeill, 1992; Vigliocco et al., 2014). Interesting to note is that our complementary CBP analysis showed that iconic gestures had long-lasting effects on processing, up to 1,000 ms post-target word. This goes considerably beyond the typical N400 window and further underlines the profound effect that co-speech gestures seem to have on semantic processing in human communication. One avenue for future research would be to zoom in on the “ripple effects” that iconic gestures have during processing longer stretches of speech. Given the long-lasting ERP effects of iconic gestures on target word comprehension in the present study (i.e., extending well beyond target offset), it is conceivable, yet untested, that they have global facilitatory effects on discourse comprehension—amounting to more than the sum of facilitatory processing of individual target words.

Complementing the literature-based N400 analysis, our exploratory CBP analysis further revealed an interaction between discourse predictability and gesture type (in addition to the main effects both predictors had). This interaction was significant during the N400 window and was distributed over right frontal electrodes. Post-hoc comparisons showed that predictable target words paired with iconic gestures elicited more positive amplitudes than predictable target words paired with control movements. Moreover, we observed a difference between iconic and control movements when presented in nonpredictable discourses. These results align with those of the planned comparisons that we conducted in our N400 analysis and suggest that iconic—compared with control—gestures had a larger effect on target word processing when presented in predictable compared with nonpredictable discourses. In fact, the pattern suggests that during discourse processing language users combine information derived from the spoken predictive input with information derived from viewing meaningful gestures and that the positive effects of both information sources add up to facilitate comprehension.

We further conjecture that participants did not attempt (at least not to a strong extent) to assign meaning to the control movements, which—as the iconic gestures—featured biological motion (e.g., hand movements), such as scratching or self-adaptors. If participants had assigned meaning to the control movements and subsequently mapped that meaning onto the meaning of the unfolding spoken target, the resulting mismatch would be reflected in large ERP differences between both conditions and should be particularly evident in the nonpredictable discourses where gesture-target integration is not influenced by predictive discourse cues, according to our cloze probability measures. Instead, the data appear to provide evidence for language users’ ability to segregate noncommunicative manual movements rather efficiently, without them incurring a processing disadvantage. Interestingly, this contrasts with the perception of beats (nonsemantic gestures indicative of emphasis), which do seem to incur a processing cost and can lead to interference (Zhang et al., 2021).

The present study advances our understanding of the relationship between linguistic predictability and iconic gestures by—among others—showing that both factors have additive positive effects on gesture-speech integration. The present experiment also complements the study by Zhang et al. (2021), who showed modulatory influences of meaningful gestures on words embedded in sentences that were extracted from spontaneous speech corpora. However, their study showed the greatest benefit of meaningful gestures for words with high surprisal values (i.e., words of low predictability). That is, the presence of iconic gestures made less predictable words more predictable and mitigated the processing costs associated with comprehending words with lower predictability. In the present study, we observed that words preceded by highly predictive discourses received a larger processing advantage compared with the same words preceded by nonpredictive discourses. While both studies may appear quite similar, there were conceptual and methodological differences, which likely gave rise to discrepant results. Most importantly, while Zhang et al. (2021) investigated moment-by-moment modulatory influences of iconic gestures by modelling the N400 amplitudes of multiple content words in a sentence, we used a different paradigm, which focused on how two-sentence predictive discourses modulate the integration of iconic gestures with target words in discourse-final position. Our paradigm was aimed at simulating discourses where predictable linguistic cues conspire to lead up to a target word. Furthermore, Zhang et al. (2021) operationalized a word’s predictability (i.e., surprisal) as bigrams, which—compared with our cloze probability measure—only takes a small portion of discourse context into account. Moreover, the stimuli in Zhang and colleagues’ study featured multiple visual cues, such as mouth informativeness and beat gestures, in addition to iconic gestures. As highlighted by the authors themselves, multimodal discourse comprehension is characterized by complex interactions between verbal and nonverbal cues. It is unclear how the weighting of the various cues contributed to the pattern of results—in particular when relating the results to the present study where the nature of the visual stimuli was confined to iconic and control movements. One possibility is that the paradigm used here drove participants towards a mode of processing were information derived from meaningful gestures and discourse cues are weighted quite strongly, which in turn enhanced gesture-speech integration. Future research could explore the interaction between multiple visual cues in discourses that lead up to predictable and nonpredictable target words by integrating parts of both experimental paradigms.

The notion that highly constraining discourse contexts facilitate multimodal integration by making upcoming words (even) more predictable and gestures more interpretable is in line with theoretical accounts where comprehenders exploit predictive cues in the spoken input to activate generalized event knowledge. Comprehenders may use the event knowledge to generate predictions about people, objects, and other entities that are likely to occur in the described event (Hintz et al., 2020; Metusalem et al., 2012). Our finding that processing was facilitated most in the predictable discourse-iconic gesture condition suggests that these predictions are rather specific, including visual information about the upcoming target words (Rommers et al., 2013; Wu & Coulson, 2011). That is, upon encountering the spoken target word, listeners had already activated features that were part of the target’s visual representation, which facilitated mapping information derived from seeing the iconic gesture onto information derived from listening to the predictable discourse context. Such an account also fits well with previous experimental work by Wu and Coulson (2007), who tested the potential of iconic gestures to prime semantic concepts. The authors presented silent video clips of iconic gestures and measured the EEG response to subsequent target words, semantically related or unrelated to the gesture. The former elicited less negative ERPs during the N400 window. Thus, although the stroke of iconic gestures preceded the onset of the spoken target by only 130 ms in the present study, this lag might have been sufficient to give a head start to gesture-speech integration, especially in the predictable discourse condition, where (some of) the gesture’s visual features were already activated.

Taken together, the present results fit well with a recent theory on predictive processing by Huettig et al. (2022). Concerning the mechanisms underlying prediction, Huettig et al. differentiate between prediction relying on *within-item* (pre-)activation (e.g., hearing the beginning of a word preactivates information about the remainder of that word at multiple levels of representation) and *between-item* (pre-)activation (activation of an item at one or multiple levels of representation spreads to associated items). In their framework, prediction (or facilitated integration) is assumed to be a natural by-product of the structure of the mental lexicon, where activation of connections between levels of word representations (within-item preactivation) and activation of connections between associated items (between-item preactivation) naturally result in (pre-)activation of interconnected information—including visual representations, such as gestures. That is, contexts in which iconic gestures are available provide the opportunity to access lexical representations via multimodal routes, which facilitates processing when no reliable discourse information is available (nonpredictable discourse-iconic gesture condition) or further enhance processing when there are linguistic cues available that allow for predictions about upcoming words (predictable discourse-iconic gesture condition).

## Conclusions

Together with other recent studies (Fritz et al., 2021; Holle & Gunter, 2007; Zhang et al., 2021), the present study builds on previous work by moving beyond paradigms at the single word or sentence level by using prose passages consisting of two consecutive sentence stimuli. This is still far removed from naturalistic language use of course, but it allows us to move one step further toward capturing some of the core features of discourse, such as the semantic constraint preceding discourse can exert and its effect on word predictability. Nevertheless, one important step for future research is to embed multimodal processing research into paradigms that better capture communication in social interaction, more naturalistic behavior, including the rich environment of other visual signals, which can modulate iconic gesture processing (Holler et al., 2015; 2014; Obermeier et al., 2015), and naturally and spontaneously produced rather than acted iconic gestures.

## Footnotes

1 We note that the present study relied on a mixed design with discourse predictability manipulated between participants. Although a within-participants design is often preferable to a between-participants design to mitigate the effects of idiosyncratic differences between individuals, our planned analyses revealed main effects of within- *and* between-participants manipulations as well as an interaction between both. To corroborate the stability of the observed effects, future research could replicate the present study using a fully-crossed within-participants design.
